# Effects of continuous isomaltulose-containing gummy intake on interstitial glucose and salivary hormones during an 18-hole golf round: a randomized, double-blind controlled pilot study

**DOI:** 10.1080/15502783.2026.2723225

**Published:** 2026-09-01

**Authors:** Yosuke Nagashima, Kiyohiro Ehara, Yoshitomo Ehara, Yusuke Maruyama, Ayana Mitsume, Yuhei Uchikoba, Junya Fukuda, Shigeru Mineo

**Affiliations:** a Department of Health Science, Musashigaoka Junior College, Saitama, Japan; b Department of Education, Tamagawa University, Tokyo, Japan; c College of Sport and Wellness, Rikkyo University, Saitama, Japan; d Candy, Dessert & Chilled Product Development Section, Second Product Development Department, Bourbon Corporation, Niigata, Japan; e Nutraceuticals Science Laboratory, Advanced Research Institutes, Bourbon Corporation, Niigata, Japan

**Keywords:** Golf, isomaltulose, glycemic variability, DHEAS, testosterone, subjective alertness

## Abstract

**Background:**

Golf is a prolonged, moderate-intensity sport requiring sustained physiological stability to manage cumulative stress and maintain performance. Although carbohydrate intake is commonly used to reduce fatigue, rapidly absorbed sugar–induced rapid blood glucose fluctuations may induce volatile arousal and latent metabolic stress. Isomaltulose, a slow-digesting disaccharide, provides a steadier glucose supply compared with sucrose. This exploratory pilot study examined the effects of isomaltulose intake on physiological stress markers, glycemic dynamics, and subjective responses during a competitive 18-hole golf round.

**Methods:**

Twenty-three male collegiate golfers were randomized to either the isomaltulose group (ISO; *n* = 12) or the sucrose group (CON; *n* = 11) in a double-blind controlled trial. Participants consumed gummies containing isomaltulose or sucrose immediately after each hole (12.1 g carbohydrate per hole; total carbohydrate intake: 217.5 g). Primary outcomes were salivary stress markers [cortisol, testosterone, and dehydroepiandrosterone sulfate (DHEAS)] levels. Secondary outcomes included interstitial glucose concentration measured via continuous glucose monitoring, subjective assessments (i.e. sleepiness, relaxation, and concentration), and golf performance (18-hole score). Between-group comparisons at each time point were conducted using planned Welch’s t-tests.

**Results:**

No significant between-group differences were observed for 18-hole score (*p* = 0.38) or mean interstitial glucose concentration (*p* = 0.20). However, exploratory analyses revealed distinct hormonal variations; salivary DHEAS and testosterone levels were higher in the ISO group during the latter half of the round (*p* < 0.05), whereas both declined in the CON group. Regarding glycemic variability, the ISO group demonstrated a more stable glucose profile with a medium effect size for lower standard deviation (ISO: 14.7 ± 1.9 vs. CON: 16.7 ± 4.6 mg/dL; *d* = 0.58), although this difference was not significant. Conversely, subjective outcomes diverged; the CON group reported significantly greater subjective arousal (wakefulness and relaxation) (*p* < 0.01) relative to the ISO group.

**Conclusions:**

In conclusion, continuous intake of isomaltulose-containing gummies during an 18-hole golf round was associated with differences in selected physiological markers, including DHEAS and testosterone concentrations. However, these findings were not accompanied by improvements in objective golf performance outcomes compared with sucrose-containing gummies. Isomaltulose may influence glycemic dynamics and hormonal responses during prolonged golf play; however, the practical significance of these effects remains exploratory. Further studies with larger sample sizes and appropriate repeated-measures frameworks are needed to determine whether such physiological changes translate into meaningful performance or recovery benefits.

## 1. Introduction

Golf involves intermittent physical exertion combined with sustained high-level cognitive demands over a prolonged duration (~4–5 h per round) [1,2]. Unlike continuous endurance sports, golfers must repeatedly alternate between physical execution and precision cognitive tasks, including green reading and course management. Although the metabolic cost of golf is generally moderate [3], the extended duration requires appropriate nutritional strategies to manage cumulative physiological stress and maintain metabolic stability, rather than simply preventing caloric depletion [4–6].

Carbohydrates serve as the primary energy source for skeletal muscle and are a key substrate for the brain, making them essential for competitive golfers [5]. However, the absorption kinetics of carbohydrates consumed during play is a major consideration [7]. Rapid blood glucose fluctuations induced by rapidly absorbed sugars can result in reactive hypoglycemia or metabolic instability. This condition potentially impairs the fine motor control required for precise play, such as putting [4]. In contrast, isomaltulose (Palatinose™), a slow-digesting structural isomer of sucrose, is hydrolysed and absorbed slowly [8], providing a steadier energy supply without pronounced glycemic fluctuations [8,9].

In a previous study, we showed that continuous carbohydrate intake via gummies maintained interstitial glucose concentrations and reduced subjective fatigue [10]. In a subsequent study, we hypothesised that stable glycemia from isomaltulose further reduces subjective fatigue compared with sucrose; however, no significant differences were detected in self-reported ratings among competitive collegiate golfers [9]. Notably, subjective assessment does not always reflect underlying physiological status. Prior research has reported a dissociation between perceived effort and objective physiological stress, particularly when nutritional interventions induce acute neural excitation or activate the reward system [11,12]. For instance, carbohydrate mouth rinsing has been shown to activate the brain’s reward system and reduce perceived exertion without altering systemic metabolic availability [11]. Moreover, salivary hormones and stress markers, including cortisol and testosterone, correlate with golf performance [13,14], indicating that objective biochemical indices may be more sensitive indicators of stress relative to self-report measures.

Accordingly, reliance on subjective measures alone may obscure the potential latent physiological benefits of isomaltulose. To address this limitation, this study focuses on objective physiological stress markers: the salivary hormones dehydroepiandrosterone sulphate (DHEAS), testosterone, and cortisol. Urinary melatonin metabolites were also assessed to evaluate nocturnal recovery, based on evidence that daytime metabolic instability can prolong physiological arousal and impair subsequent sleep quality [15,16]. By prioritising these biomarkers, this exploratory pilot study aimed to bridge the gap between subjective perception and physiological status under real-world, outdoor sport conditions. We hypothesised that continuous isomaltulose intake would maintain stable glycemia and better sustain the salivary endocrine profile (DHEAS and testosterone) more effectively than sucrose, thereby mitigating cumulative physiological stress. In summary, this work reports the preliminary effects of continuous isomaltulose intake on interstitial glucose dynamics, salivary stress markers, and subjective states during a competitive 18-hole golf round.

## 2. Materials and methods

### 2.1. Study design and randomisation

**Figure 1.**
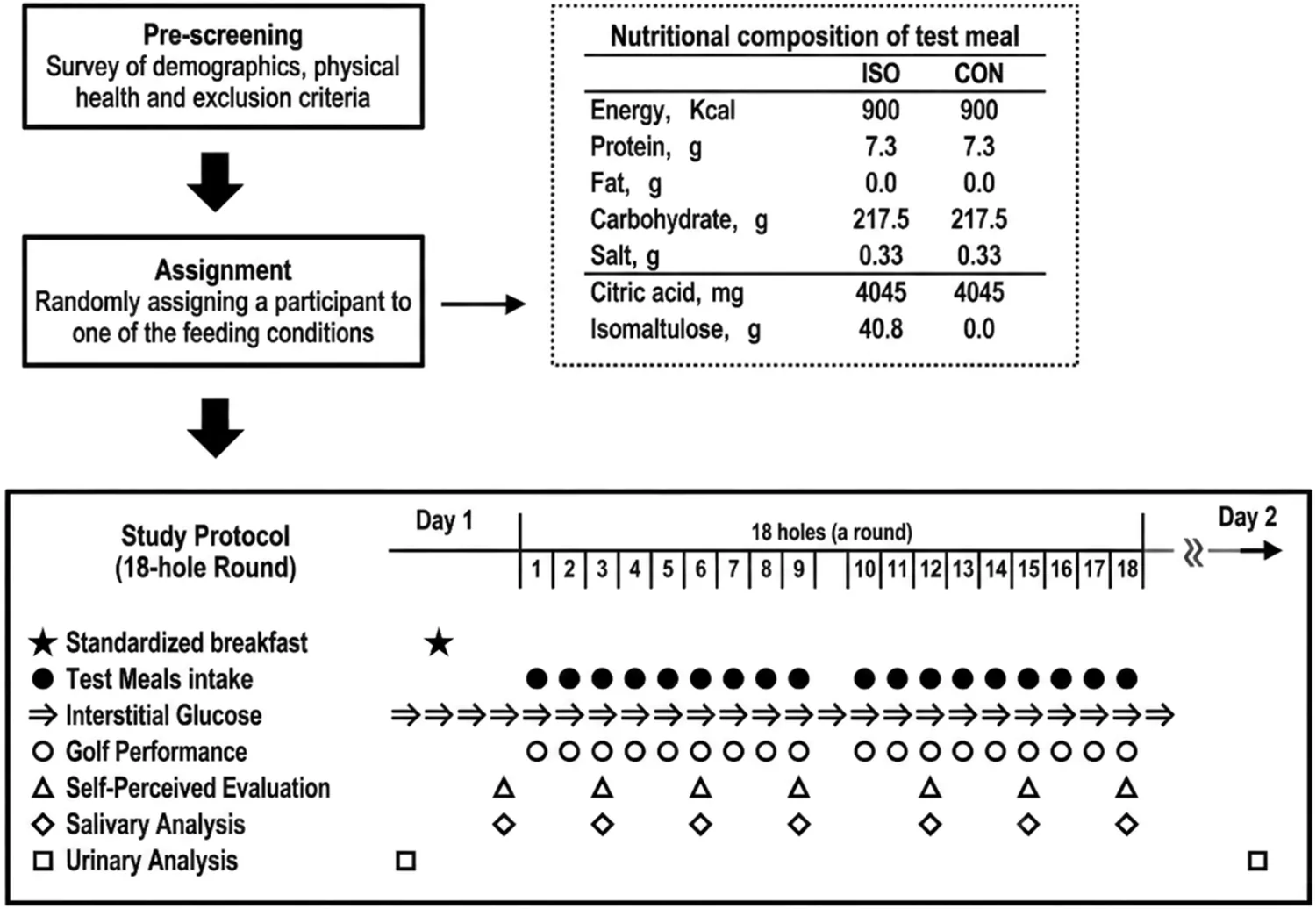
Schematic representation of the experimental protocol. The study timeline began at 06:00 with application of continuous glucose monitoring sensors. Participants consumed a standardised breakfast ~2 h before the round began. The 18-hole round included a 40-min break after the first 9 holes. Test gummies (isomaltulose or sucrose) were ingested immediately after completing each hole. Saliva samples and subjective assessments (visual analogue scale and Stanford sleepiness scale) were obtained at seven time points: baseline (hole 0) and after every three holes (holes 3, 6, 9, 12, 15, and 18). Urine samples were collected before the round and immediately after completion.

A randomised, double-blind, parallel-group design was employed (Figure 1). To minimise heterogeneity, participants were allocated via stratified block randomisation based on 2024 competitive performance and body weight to either the isomaltulose (ISO) or sucrose (CON) gummy group.

### 2.2. Ethics and compliance

Conducted in accordance with the Declaration of Helsinki, the protocol was approved by the Musashigaoka Junior College Ethics Committee (Approval No. 3; July 22, 2024) and registered in the UMIN-CTR (ID: UMIN000055255). Electronic informed consent was obtained from all participants following an online explanation of the study.

### 2.3. Participants

Twenty-four male golfers were recruited from Division B of the Kanto University Golf Federation. A priori power analysis (JMP v18.1.0; Cohen’s *d* ≥ 0.80 [9], *α* = 0.05, power = 80%) indicated a minimum sample of 22; 24 were enroled to account for attrition. Inclusion criteria were: (1) healthy male collegiate golfers, (2) active competitive participation, and (3) no performance-limiting musculoskeletal disorders. Exclusion criteria included: (1) ingredient allergies, (2) medications affecting glucose metabolism, or (3) investigator-determined unsuitability. Baseline characteristics were collected to facilitate stratified randomisation.

### 2.4. Experimental procedures

The study was conducted at Ome Golf Club (Tokyo, Japan) on September 10, 2024. Participants were instructed to abstain from alcohol for 12 hours and fast overnight (water permitted) prior to the trial. On the trial day (arrival at 05:20), a continuous glucose monitoring (CGM) device was attached at approximately 05:30. Between 06:40 and 07:10, participants consumed a standardised breakfast consisting of rice, miso soup, grilled salmon, natto, and a raw egg. Nutrient analysis based on the Standard Tables of Food Composition in Japan (2020) indicated an energy content of 580 kcal (comprising 22.2% protein, 17.7% fat, and 63.9% carbohydrates). The total carbohydrate content (92.8 g; ~1.38 g/kg) met the ACSM pre-exercise guidelines [17]. Following a warm-up, play commenced between 07:53 and 08:28, including a 40-min half-time rest. Test gummies were consumed after each hole, and participants were instructed to chew them approximately 30 times. Water intake was standardised to 3000 mL, and the consumption of other foods or fluids was prohibited. The mean active round duration was 5 hours and 4 minutes. Hourly carbohydrate intake was calculated as 43.8 g/h for the ISO group and 43.5 g/h for the CON group. To mimic real-world tournament conditions, participants were offered performance-based incentives [18].

### 2.5. Test foods

Test foods, provided by Bourbon Corporation (Niigata, Japan), comprised two gummy products. To ensure double blinding, gummies were visually and flavour-identical, packaged in opaque bags. The ISO group consumed isomaltulose gummies; the CON group, sucrose gummies (Figure 1). Total energy and macronutrients were matched (900 kcal; 217.5 g carbohydrate). The ISO formulation provided 40.8 g of isomaltulose (~19% of total carbohydrates), the maximum feasible for gummy production. Carbohydrate intake rate (~43.5 g/h) met ACSM guidelines (30–60 g/h for exercise > 1 h) [17,19].

### 2.6. Measurements

#### 2.6.1. Interstitial glucose monitoring

Interstitial glucose was continuously measured using a factory-calibrated flash glucose monitoring system (FreeStyle Libre Pro, Abbott Diabetes Care). A sensor was placed on the nondominant upper arm to record glucose levels every 15 min. This device exhibits a mean absolute relative difference of 11.4% compared with venous blood measurements [20]. Participants were blinded to the real-time glucose data throughout the study.

#### 2.6.2. Biochemical analyses salivary hormones and stress markers

Saliva samples were collected via the passive drool method using short polypropylene straws and storage tubes at seven distinct time points: baseline (preround) and immediately after completion of every third hole (holes 3, 6, 9, 12, 15, and 18; Figure 1). This sampling interval (approximately every 45–60 min) was chosen to capture dynamic physiological fluctuations while minimising disruption to the flow of competitive play. This method was selected to eliminate assay interference with testosterone commonly associated with cotton or synthetic swabs [21]. To prevent contamination, sampling was performed before water or gummy intake at each time point. Participants allowed saliva to pool in the mouth and drooled through the straw directly into the collection vial. This approach is considered the gold standard for salivary steroid analysis as it ensures sample integrity without introducing materials that may alter hormone concentrations [21]. Sample collection was supervised by research staff to ensure protocol adherence. Immediately after collection, samples were stored in portable cooler boxes and maintained at around 4°C during the round to limit degradation [22]. All samples were then transported to Rikkyo University, Tokyo, Japan, under frozen conditions for biochemical analysis. All saliva samples were stored at −80°C until sample preparation and analysis.

Saliva samples were thawed on ice prior to analysis. Methanol (400 µL) was added to 1.5-mL microcentrifuge tubes, followed by the addition of 100 µL of saliva. The mixture was vortexed for 5 min and centrifuged at 14,000 rpm for 10 min at 4°C. Subsequently, 100 µL of the supernatant was transferred to a centrifugal filter unit (Ultrafree-MC-GV, PVDF 0.22 µm; Merck Millipore, Darmstadt, Germany) and centrifuged at 10,000 rpm for 2 min at 4°C. The filtrate was transferred to LC–MS sample vials and subjected to LC–MS/MS analysis.

#### 2.6.3. Urinary melatonin metabolites

Urine samples were collected at two time points (Figure 1): (1) at baseline on the trial morning (day 1 at ~05:30) and (2) the following morning (day 2 at 07:00–08:00). Baseline samples obtained at the study site were immediately placed in cooler boxes at 4°C until freezing. For Day 2 collection, participants obtained urine samples at home, immediately froze them, and transported them using a frozen courier service. All urine samples were stored at −80 °C in the laboratory until analysis.

Urinary 6-sulfatoxymelatonin (6-SMEL) was measured as an indicator of nocturnal melatonin secretion using an LC–MS/MS. Urinary 6-SMEL is an established noninvasive surrogate marker reflecting total nocturnal plasma melatonin output [23]. Urine samples were prepared using the same procedure as saliva samples. Briefly, urine samples were thawed on ice, and 100 µL of urine was mixed with 400 µL of methanol. After vortexing for 5 min, samples were centrifuged at 14,000 rpm for 10 min at 4°C. An aliquot of 100 µL of the supernatant was transferred to a centrifugal filter unit and centrifuged at 10,000 rpm for 2 min at 4°C. The filtrate was collected in LC–MS sample vials for subsequent analysis.

To adjust for urine dilution, urinary creatinine concentrations were measured concurrently using a colorimetric assay (LabAssay Creatinine, FUJIFILM Wako Pure Chemical Corporation, Osaka, Japan). The 6-SMEL concentration was normalised to creatinine and expressed as µg/mg Cre. This normalisation approach is standard in field research to correct for hydration status and urine volume variability.

#### 2.6.4. LC–MS/MS analysis

Cortisol, testosterone, dehydroepiandrosterone sulphate (DHEAS), and 6-sulfatoxymelatonin (6-SMEL) were quantified using liquid chromatography–tandem mass spectrometry (LC–MS/MS). Samples (10 µL) were injected into a liquid chromatography system (AC30AD; Shimadzu Corporation) equipped with a C18 column (2.0 I.D. × 150 mm, 3 µm; TSKgel ODS-100V, Tosoh Corporation, Tokyo, Japan). The mobile phase consisted of 10 µM ammonium acetate in 0.05% (v/v) acetic acid with methanol as the organic solvent. A linear gradient was applied from 5% to 50% methanol over 20 min, followed by a wash with 100% methanol for 10 min. The flow rate was set at 0.3 mL/min. The column oven and autosampler temperatures were maintained at 25°C and 4°C, respectively. Analytes were detected using a triple quadrupole mass spectrometer (LCMS-8050; Shimadzu Corporation, Kyoto, Japan) operated in multiple reaction monitoring (MRM) mode with electrospray ionisation (ESI). The monitored MRM transitions (m/z) were 363.0 ⟶ 121.1 for cortisol, 289.2 ⟶ 109.1 for testosterone, 367.2 ⟶ 97.0 for DHEAS, and 327.0 ⟶ 161.0 for 6-SMEL.

### 2.7. Subjective assessments and golf performance

#### 2.7.1. Subjective assessments

Self-perceived physiological and psychological states were assessed at seven time points: baseline and immediately after completion of every third hole (holes 3, 6, 9, 12, 15, and 18; Figure 1). Measurements were obtained using a visual analogue scale (VAS) [24], in which participants marked their state on a 100-mm horizontal line anchored by “Not at all” (0 mm) and “Extremely” (100 mm). The evaluated domains were “Relaxation,” “Concentration,” and “Fatigue.” Additionally, subjective sleepiness was assessed using the Stanford sleepiness scale (SSS), a 7-point Likert scale ranging from “Feeling active, vital, alert” (1) to “No longer fighting sleep” (7) [25]. All subjective assessments were performed concurrently with saliva sampling to minimise interruption of play.

#### 2.7.2. Golf performance

Golf performance was quantified using gross score, defined as the total number of strokes for each hole. As indicated in the experimental timeline (Figure 1), participants recorded scores immediately after completing each hole. To ensure data accuracy, scores were cross-checked by playing partners acting as markers in accordance with the official Rules of Golf. This mutual verification approach is widely used in competitive golf research to guarantee ecological validity and measurement reliability [4,7]. The final 18-hole score was calculated as the sum of strokes across all holes.

### 2.8. Statistical analysis

All data are presented as means ± standard deviations (SDs). Statistical analyses were performed using JMP Pro v18.2.2 (SAS Institute Inc., Cary, NC, USA). Before analysis, the Smirnov–Grubbs test was applied to identify and exclude outliers at each measurement point [26]. Baseline characteristics were compared using Welch’s t-test for continuous variables and Fisher’s exact test for categorical variables to confirm the absence of significant pretrial group differences.

Welch’s t-test was employed for between-group comparisons at each time point. Although repeated-measures analysis of variance is commonly applied to time-course data, it was not applied here due to sample size constraints (*n* = 23). In small-sample field research, multi-factor models can severely restrict degrees of freedom, inflating the risk of Type II errors (false negatives) and masking true physiological trends. Furthermore, significant baseline imbalances occurred by chance (e.g. salivary cortisol, *p* = 0.026); applying global interaction models to such data can distort longitudinal interpretations. Therefore, we selected planned comparisons with Welch’s t-test based on an *a priori* hypothesis. Given the slow absorption kinetics of isomaltulose, we hypothesised that physiological differences would not be uniform but would emerge primarily during the latter half of the round. Centred on this exploratory pilot framework, point-by-point comparisons were considered more appropriate for detecting time-dependent responses. Additionally, Welch's t-test was selected to accommodate heteroscedasticity unequal variances, known as heteroscedasticity, frequently observed in field data, a method recommended for controlling Type I error rates without over-penalising the analysis under small-sample constraints [27,28].

To limit inflation of Type II errors, or false negatives, and avoid masking meaningful trends, strict corrections for multiple comparisons (e.g. Bonferroni adjustment) were not applied. This approach aligns with the notion that in applied, exploratory field studies, such corrections may overly penalise the detection of biologically relevant effects [29,30]. Instead, interpretation emphasised effect magnitude alongside statistical significance. Effect sizes were calculated using Cohen’s *d* and classified as small (0.20 ≤ *d* < 0.50), medium (0.50 ≤ *d* < 0.80), and large (*d* ≥ 0.80) [31]. All tests were two-tailed, with statistical significance set at *p* < 0.05.

## 3. Results

### 3.1. Participant characteristics, golf performance, and mean interstitial glucose

Of the 24 enroled participants, one withdrew on the trial day due to acute illness. Consequently, 23 participants (ISO: *n* = 12; CON: *n* = 11) completed the study and were included in the analysis. Statistical outliers identified via the Smirnov–Grubbs test were excluded from the final dataset. Specifically, only two extreme data points were excluded: one for baseline salivary cortisol in the CON group (*n* = 1) and one for Day 2 morning urinary 6-SMEL in the ISO group (*n* = 1). Re-running the statistical analyses with these outliers included did not alter any of the significance levels or the final conclusions of the study.

Table 1 summarises participant characteristics, golf performance, and interstitial glucose profiles. No significant differences were observed in baseline anthropometrics (*p* > 0.05, *d* < 0.20) or competition level (*p* = 1.00) between groups, confirming successful randomisation.

**Table 1.** Participant characteristics and experimental outcomes.

Characteristics	ISO group (*n* = 12)	CON group (*n* = 11)	*P-*value	Cohen’s *d*
Age (years)	20.2 ± 1.2	20.2 ± 1.3	0.98	0.01
Height (cm)	173.7 ± 5.9	172.4 ± 5.4	0.58	0.23
Body mass (kg)	66.7 ± 5.4	69.5 ± 9.6	0.39	0.37
Competition level			1.00^†^	-
National	2 (25.0)	3 (18.2)		
Regional (Kanto)	9 (75.0)	9 (81.8)		
18-hole total score	75.3 ± 4.5	75.5 ± 5.5	0.91	0.05
Interstitial glucose (mg/dL)	96.8 ± 12.6	102.3 ± 5.1	0.20	0.57
Interstitial glucose SD (mg/dL)	14.7 ± 1.9	16.7 ± 4.6	0.23	0.58
Interstitial glucose CV (%)	15.4 ± 2.9	16.3 ± 4.2	0.61	0.23

Values are reported as means ± standard deviations or counts (percentages). ISO: isomaltulose-containing gummy group; CON: sucrose-containing gummy group. Continuous variables were analysed using Welch’s t-test. Effect sizes were calculated using Cohen’s *d* and interpreted as follows: small (0.20 ≤ *d* < 0.50), medium (0.50 ≤ *d* < 0.80), and large (*d* ≥ 0.80). † *p*-value calculated using Fisher’s exact test.

Total 18-hole gross scores did not differ significantly (75.3 ± 4.5 vs. 75.5 ± 5.5 strokes; *p* > 0.05, *d* = 0.05). Mean interstitial glucose concentrations were slightly lower in the ISO group (96.8 ± 12.6 mg/dL) compared with the CON group (102.3 ± 5.1 mg/dL). Although not significant (*p* = 0.20), the effect size was medium (*d* = 0.57), suggesting an exploratory trend that warrants further validation.

### 3.2. Interstitial glucose dynamics

**Figure 2.**
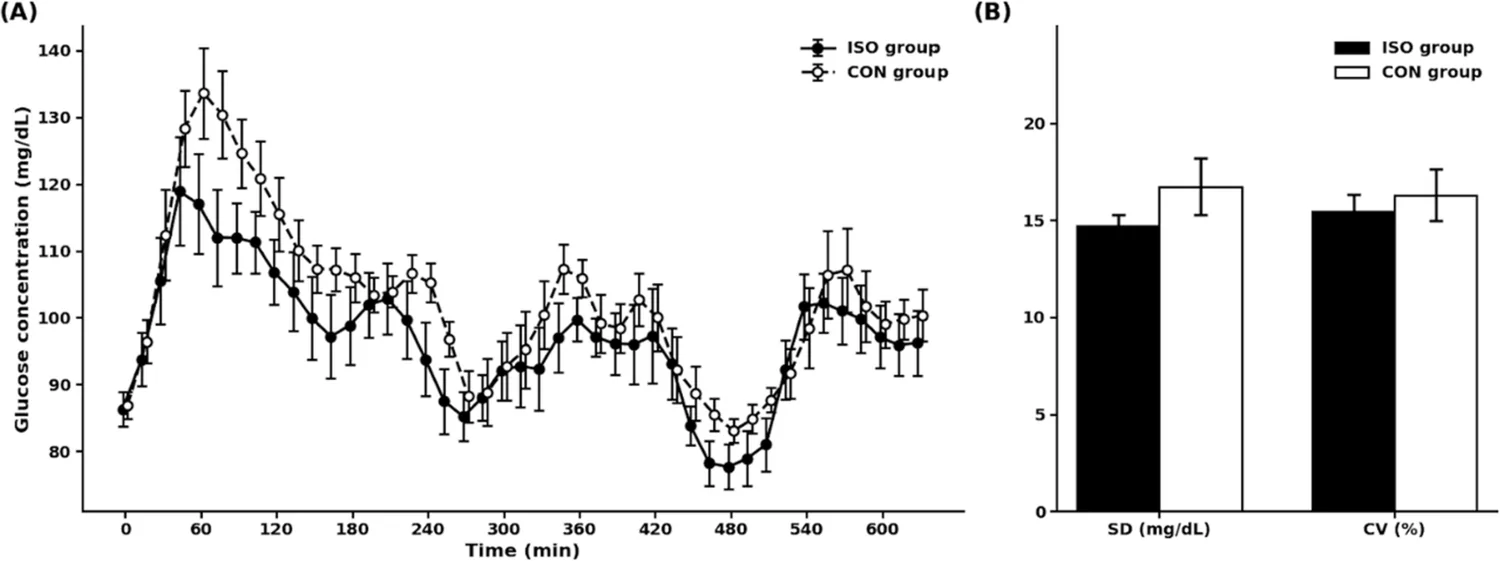
Time-course changes in interstitial glucose and glycemic variability during the 18-hole round. (A) Temporal changes in interstitial glucose concentrations, with the solid line indicating the ISO group (*n* = 12) and the dashed line indicating the CON group (*n* = 11). (B) Comparison of glycemic variability assessed by glucose standard deviation (SD) and coefficient of variation (CV). The ISO group showed a tendency toward lower absolute variability (SD) with a medium effect size (*d* = 0.58), although the difference was nonsignificant (*p* = 0.23). Relative variability (CV) also showed a small effect size (*d* = 0.23). Data are presented as means ± standard errors of the means. Abbreviations: ISO, isomaltulose-intake group (●); CON, sucrose-intake group (○).

Time-course changes in interstitial glucose levels are shown in Figure 2A. The ISO group generally maintained lower and more stable levels throughout the round compared with the CON group. Glucose concentrations in the ISO group remained largely within a stable range (80–110 mg/dL), whereas the CON group exhibited marked fluctuations with peak values exceeding 130 mg/dL. This aligns with the medium effect size observed for mean glucose concentrations.

Regarding glycemic variability (Figure 2B), the ISO group exhibited lower absolute variability (SD) compared with the CON group (14.7 ± 1.9 vs. 16.7 ± 4.6 mg/dL). Although this difference was not significant (*p* = 0.23), the effect size was medium (*d* = 0.58), suggesting a preliminary trend toward glycemic stability under these pilot conditions. In contrast, relative variability (coefficient of variation) showed a small effect (*p* = 0.61, *d* = 0.23).

### 3.3. Objective physiological stress markers

**Figure 3.**
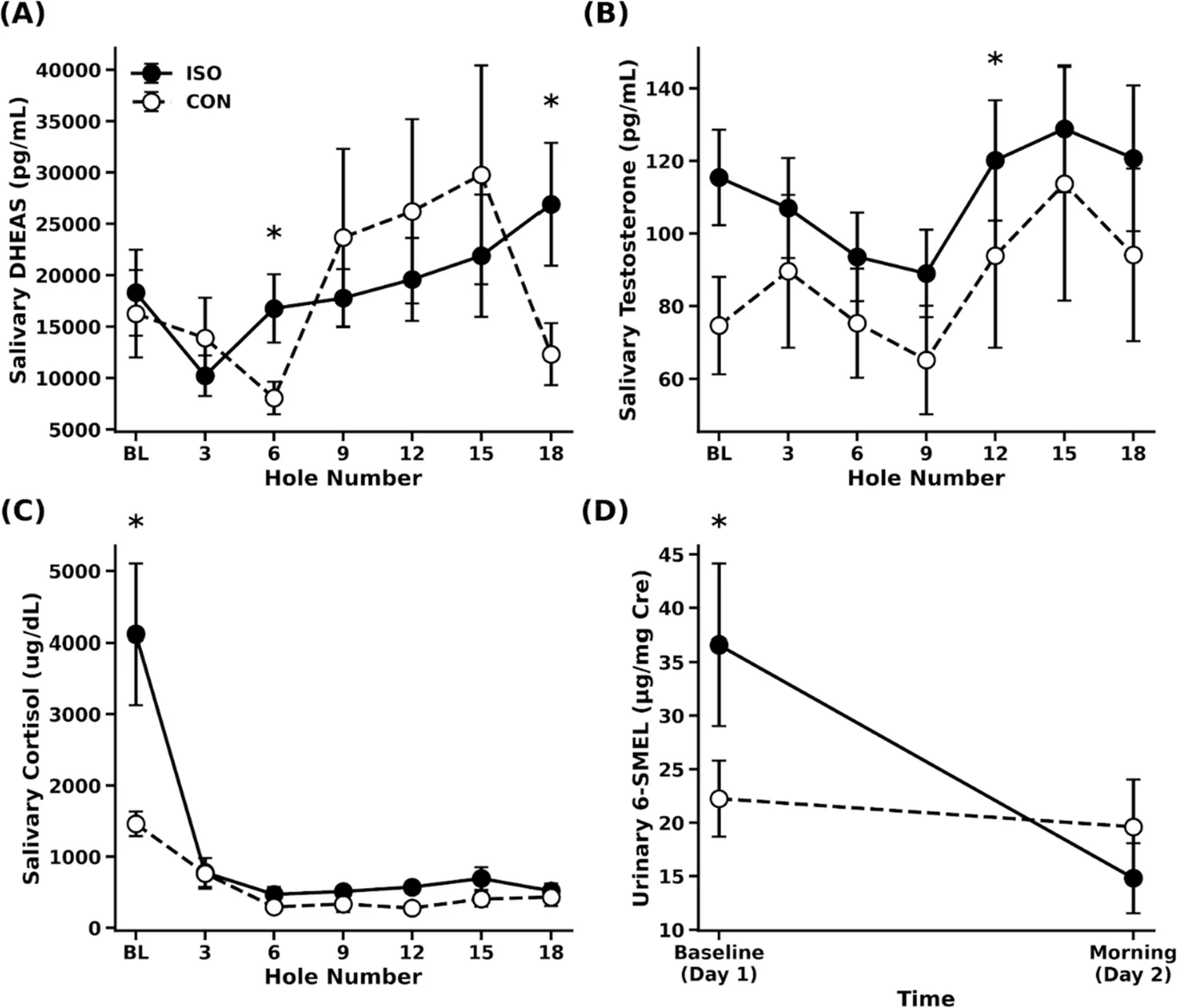
Changes in physiological stress markers and melatonin metabolism. (A) Salivary DHEAS, (B) salivary testosterone, and (C) salivary cortisol concentrations measured at every third hole. (D) Urinary 6-sulfatoxymelatonin (6-SMEL) concentrations measured at baseline (day 1) and the following morning (day 2). Values are presented as means ± standard errors of the means. Closed circles (●) denote the ISO group (*n* = 12); open circles (○) denote the CON group (*n* = 11). * indicates a significant between-group difference (*p* < 0.05). As shown in D, baseline levels of 6-SMEL were significantly higher in the ISO group, contributing to the larger apparent posttrial decrease.

Time-course changes in salivary hormones are presented in Figure 3. Salivary DHEAS concentrations (Figure 3A) were significantly elevated in the ISO group relative to the CON group after hole 6 (15,720 ± 9,826 vs. 7,637 ± 3,947 pg/mL; *p* = 0.025, *d* = 1.08) and at hole 18 (26,529 ± 18,442 vs. 12,332 ± 6,619 pg/mL; *p* = 0.027, *d* = 1.02). Similarly, salivary testosterone levels at hole 12 (Figure 3B) were significantly higher in the ISO group (105.8 ± 58.0 vs. 63.1 ± 17.2 pg/mL; *p* = 0.031, *d* = 1.00). Baseline salivary cortisol (Figure 3C) was significantly higher in the ISO group (4116 ± 3129 vs. 1461 ± 456 pg/mL; *p* = 0.026, *d* = 1.09), but no significant differences were observed between groups during the round (*p* > 0.05).

Regarding urinary melatonin metabolism (Figure 3D), baseline 6-SMEL concentrations were significantly higher in the ISO group (37.9 ± 9.4 vs. 22.2 ± 5.6 μg/mg Cre; *p* = 0.038). From day 1 to 2, 6-SMEL levels decreased in both groups, with a larger reduction observed in the ISO group (−12.4 ± 12.5 vs. −2.6 ± 11.0 μg/mg Cre; *d* = 0.83). However, absolute concentrations on day 2 did not differ significantly between groups (*p* > 0.05).

### 3.4. Subjective assessments and time-course performance

**Figure 4.**
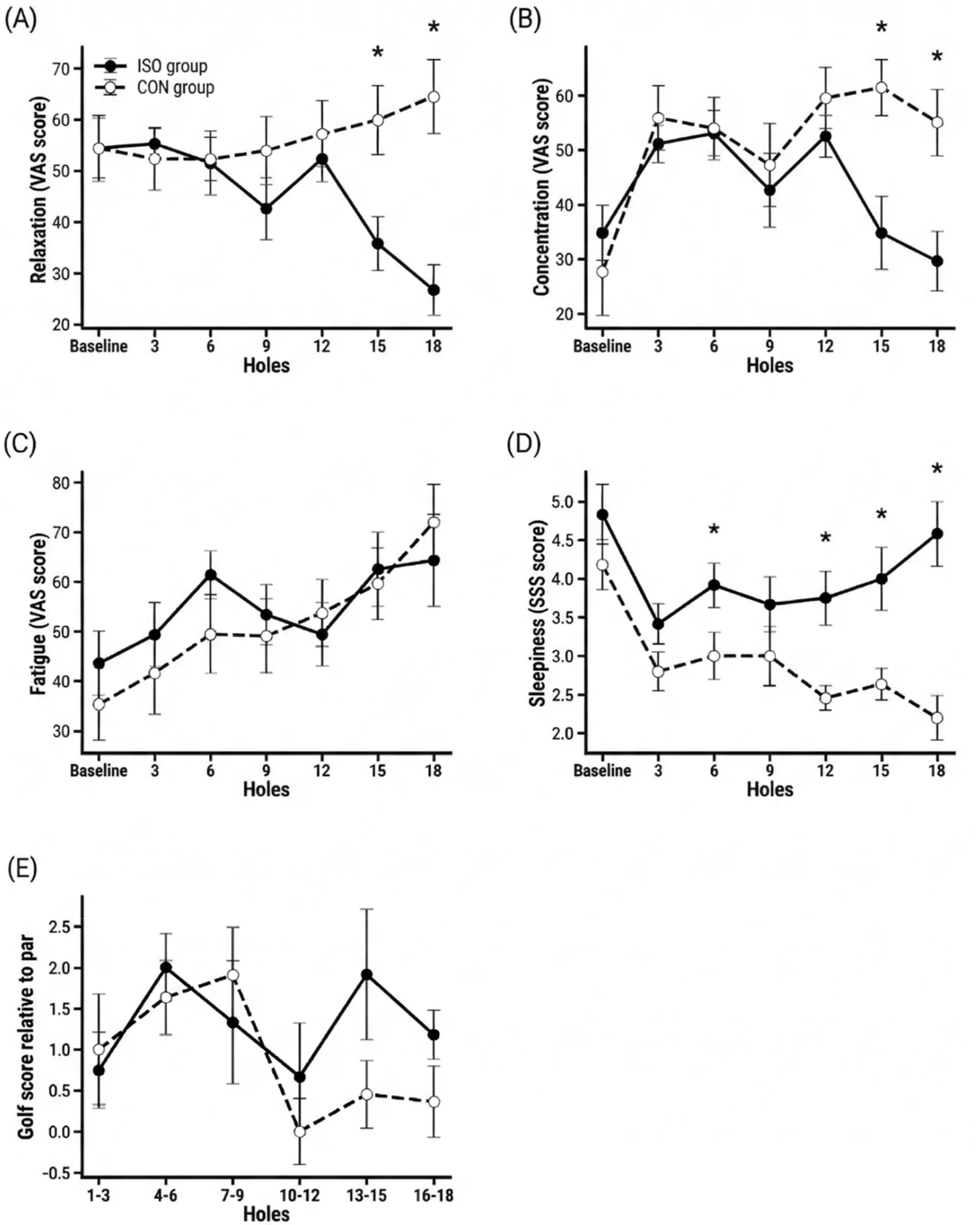
Time-course changes in subjective assessments and golf performance. (A) Relaxation, (B) concentration, and (C) fatigue assessed using the visual analogue scale. (D) Sleepiness assessed using the Stanford sleepiness scale. (E) Golf score relative to par calculated every three holes. Closed circles (●) indicate the ISO group (*n* = 12), and open circles (○) indicate the CON group (*n* = 11). Values are expressed as means ± standard errors of the means. * indicates a significant difference between groups (*p* < 0.05).

Time-course changes in subjective ratings (SSS and VAS) revealed divergent patterns between groups. In contrast to the ISO group’s stable physiological profile, the CON group maintained more favourable subjective states during the latter half of the round (Figure 4).

Regarding subjective relaxation (Figure 4A), the CON group exhibited higher scores during the final stages: significant differences were observed after holes 15 (*p* = 0.011, *d* = 1.19) and 18 (26.8 ± 17.0 vs. 64.5 ± 23.8 mm; *p* < 0.001, *d* = 1.82). Subjective concentration scores (Figure 4B) were similarly higher in the CON group after holes 15 and 18 (*p* < 0.01). However, subjective fatigue showed no significant group differences at any time point (Figure 4C).

For sleepiness (Figure 4D), a significant difference was observed at hole 6 (*p* = 0.039, *d* = 0.92). Additionally, the CON group reported significantly lower SSS scores (indicating greater wakefulness) from hole 12 onward (*p* = 0.004, *d* = 1.38), persisting at holes 15 (*p* = 0.009, *d* = 1.23) and 18 (4.6 ± 1.4 vs. 2.2 ± 0.9; *p* < 0.001, *d* = 1.97).

Despite these subjective differences, golf performance relative to par (analysed every three holes) did not differ significantly between groups at any interval (*p* > 0.05; Figure 4E).

## 4. Discussion

This study highlights a clear dissociation between subjective psychological states and objective neuroendocrine responses during a competitive golf round. Although objective golf performance and mean interstitial glucose levels were comparable between the ISO (isomaltulose gummy) and CON (sucrose gummy) groups, the underlying physiological and psychological pathways differed markedly. Subjectively, rapid sucrose availability appeared to support positive mood states, evidenced by significantly higher relaxation and concentration ratings and reduced sleepiness, particularly in the latter half of the round. This observation aligns with prior studies indicating that rapidly absorbed carbohydrates can activate the reward system and acutely enhance mood [11,32]. Specifically, the rapid influx of glucose may stimulate dopaminergic transmission in the brain’s reward system, thereby generating a transient sensation of well-being and reduced perceived effort. However, in objective terms, the ISO group displayed a superior neuroendocrine profile. Despite lower subjective arousal, ISO group participants maintained significantly higher DHEAS and testosterone levels during critical stages of the round. This paradox suggests that isomaltulose intake may influence acute endocrine responses at a sub-perceptual level, providing preliminary metabolic stability without affecting overall golf performance.

The divergence in subjective sensation likely reflects the distinct neurometabolic responses elicited by the test carbohydrates. In the CON group, the frequent intake of sucrose (a rapidly digested disaccharide) likely produced repeated blood glucose excursions. Because the brain relies on glucose as its primary energy substrate, these rapid fluctuations may have stimulated the reward system, masking perceived fatigue via acute neural excitatory peaks [11,33]. This acute stimulation may transiently facilitate motor performance and sustained attention, effectively allowing athletes to execute complex motor skills despite accumulating physiological fatigue.

The lower subjective arousal observed in the ISO group warrants consideration of a key baseline confounding factor. Notably, ISO group participants exhibited significantly higher baseline concentrations of 6-SMEL, the primary melatonin metabolite. Elevated morning 6-SMEL levels suggest that the physiological transition from sleep to wakefulness was incomplete, potentially reflecting prolonged sleep inertia [34]. Consequently, ISO group participants were likely physiologically predisposed to drowsiness on the trial day, independent of carbohydrate intervention. This indicates that the observed deficit in subjective wakefulness is at least partially attributable to baseline circadian differences rather than any metabolic lethargy or sedative effect of isomaltulose [35].

In contrast to observed lower subjective arousal, the physiological benefits of isomaltulose were evident in glycemic and neuroendocrine markers. The ISO group exhibited a more stable interstitial glucose profile with significantly lower glycemic variability relative to the CON group. This pattern aligns with the physicochemical properties of isomaltulose, which is hydrolysed and absorbed 4–5 times slower than sucrose, providing sustained glucose supply without provoking insulin spikes or reactive hypoglycemia [8,36]. This steady energy delivery likely underpins the sustained levels of DHEAS and testosterone observed in ISO group participants. Testosterone and DHEAS are highly sensitive to metabolic stress and typically decline during prolonged exertion when energy availability is limited [37,38]. In the present study, maintenance of these hormones in the ISO group, despite the physical demands of an 18-hole round, suggests that continuous glucose supply mitigated systemic energy deficits induced by both mental pressure and physical exertion. Thus, although participants’ conscious perception may have reflected lower arousal due to circadian factors, their metabolic systems demonstrated sustained neuroendocrine levels, avoiding the acute declines in DHEAS and testosterone often observed during prolonged physical exertion [39]. However, these physiological changes in selected markers did not translate into any meaningful improvements in objective golf performance outcomes, and their practical significance remains exploratory.

Unexpectedly, baseline salivary cortisol concentrations were significantly higher in the ISO group compared with the CON group. Given random group assignment, this discrepancy likely reflects inherent interindividual variability in basal neuroendocrine activity [21,40] or differences in anticipatory psychological stress prior to the round. Additionally, the cortisol awakening response, which causes a sharp rise in cortisol levels 30–45 min after waking, could have contributed if wake times varied by chance. Importantly, this baseline difference disappeared once the round began, with no significant group differences observed thereafter. This convergence suggests that continuous carbohydrate intake, regardless of source, provided sufficient metabolic support to attenuate the stress response, effectively mitigating the initial cortisol elevation.

A central insight from this study is the pronounced dissociation between subjective well-being and physiological stress status, demonstrating that subjectively “feeling good” does not equate to the absence of physiological stress. Although CON group participants reported no subjective discomfort, objective markers, such as DHEAS levels, remained significantly lower, suggesting latent physiological stress. Conversely, the ISO group was characterised by sustained glycemic stability, which effectively precluded the acute neural excitatory peaks and volatile arousal peaks associated with rapid blood glucose fluctuations [41]. Although the absence of these peaks likely contributed to lower subjective alertness in ISO group participants, the data clearly show that the associated neuroendocrine trajectories remained more stable.

Notably, the isomaltulose content in the test gummies constituted ~19% (40.8 g) of the total carbohydrate, reflecting the maximum feasible inclusion rate due to physicochemical manufacturing constraints. Despite this modest proportion, distinct variations were observed in salivary DHEAS and testosterone responses. This finding is highly encouraging and aligns with prior research showing that even partial carbohydrate blends containing isomaltulose effectively modulate metabolism [42]. Thus, substituting only a portion of sucrose with isomaltulose may suffice to influence endocrine trajectories and mitigate acute physiological stress during competitive sports without requiring complete dietary modification.

From a practical perspective, these results offer valuable guidance for nutritional strategies not only in golf but also in other duration-dependent activities characterised by moderate intensity, mirroring real-life physical demands. Athletes and coaches should recognise that subjective alertness does not always reflect physiological recovery or the underlying neuroendocrine responses. Although rapid-acting carbohydrates, such as sucrose, can transiently elevate mood and mask perceived fatigue via acute neural excitatory peaks, relying solely on them may leave athletes vulnerable to latent stress accumulation. In contrast, slow-digesting carbohydrates, such as isomaltulose, function as a metabolic stabiliser, effectively suppressing catabolic stress responses and preventing cellular energy depletion even without relying on neural arousal [43]. Therefore, for prolonged activity where maintaining physical condition is critical, prioritising slow-digesting carbohydrates (e.g. isomaltulose) or strategically combining carbohydrate sources to balance mood and metabolic stability may be optimal strategies for mitigating cumulative physiological stress and supporting long-term performance [44].

The present study has several limitations that provide avenues for future research. First, our sample size (*n* = 23) was logistically limited by the demanding nature of real-world, outdoor 18-hole golf field testing, meaning strict multiple-comparison adjustments were not applied to this exploratory pilot framework. Consequently, while the observed large effect sizes suggest sufficient sensitivity for detecting preliminary psychophysiological trends, they may be underpowered for identifying subtle changes in objective golf performance. Furthermore, significant baseline imbalances occurred by chance—specifically in salivary cortisol (*p* = 0.026) and melatonin metabolism—which can confound longitudinal global interpretations in small-sample trials. Therefore, future definitive studies should recruit larger cohorts to ensure adequate statistical power, allowing baseline values to be included as covariates within a repeated-measures ANCOVA framework. Second, daily variability in sleep quality and circadian rhythm can influence golf performance and physiological recovery. In the present study, urinary analysis revealed a significant baseline imbalance: even after excluding statistical outliers (Smirnov–Grubbs test, *p* < 0.05), baseline 6-SMEL levels were higher in the ISO group than in the CON group (*p* = 0.038). The larger decrease in 6-SMEL observed in the ISO group likely reflects physiological circadian decline and regression to the mean rather than an intervention effect [45], highlighting a confounding factor that limits conclusions regarding isomaltulose’s impact on nocturnal melatonin secretion. Third, methodological limitations regarding glucose monitoring must be acknowledged. The CGM system measures interstitial rather than blood glucose, introducing a physiological time lag [46] and typically attenuating peak values relative to direct blood sampling; thus, our results may underestimate the magnitude of the sucrose-induced acute glycemic spike [47]. Although CGM allows continuous assessment without interrupting play, future studies requiring precise pharmacokinetic data should incorporate frequent venous blood sampling. Fourth, although we hypothesised that the dissociation between physiological stability and subjective arousal was due to differences in sucrose-induced acute neural stimulation, neurophysiological measures were not employed. Consequently, the hypothesis that this neural mechanism “masked” perceived fatigue remains speculative. Incorporating electroencephalography or other neuroimaging techniques could directly assess brain activity and clarify underlying neural mechanisms [48]. Fifth, although the field setting enhanced ecological validity, controlling environmental factors was challenging [49]. Additionally, participants’ wake-up times were not strictly standardised, potentially influencing baseline cortisol via the cortisol awakening response. Furthermore, practice-round conditions differ psychologically from official competition; future research should replicate these protocols in controlled indoor simulators to clarify physiological mechanisms or target official competitive rounds, including multiday tournaments, to assess efficacy under conditions involving high pressure and cumulative fatigue. Sixth, isomaltulose supplied only 40.8 g of the 217.5 g total carbohydrate intake (~19%) due to gummy manufacturing constraints. Given that over 80% of the carbohydrate matrix was identical between groups, attributing the observed neuroendocrine differences solely to isomaltulose warrants caution as this mixed formulation represents a confounding factor. Seventh, we did not apply multi-factor interaction models or aggregate global metrics. Because our primary hypothesis targeted point-by-point physiological changes during the late phase of the 18-hole round, global models could smooth out these acute, time-dependent variations and mask late-stage intervention trends. However, this discrete statistical approach increases the risk of Type I errors, and these isolated findings must be interpreted strictly within an exploratory framework. Finally, although 6-SMEL reliably indicates melatonin output, it does not quantify sleep quality. Future studies should therefore integrate objective measures, such as actigraphy or polysomnography, to assess sleep efficiency and latency for a comprehensive evaluation of recovery.

## 5. Conclusion

In conclusion, continuous intake of isomaltulose-containing gummies during an 18-hole golf round was associated with differences in selected physiological markers, including DHEAS and testosterone concentrations. However, these findings were not accompanied by improvements in objective golf performance outcomes compared with sucrose-containing gummies. The practical significance of these physiological variations remains exploratory, and future studies with larger sample sizes and appropriate repeated-measures frameworks are needed to validate these preliminary trends.

## Data Availability

The datasets used and/or analysed during the current study are available from the corresponding author on reasonable request.

## References

[1] Broman G , Johnsson L , Kaijser L . Golf: a high intensity interval activity for elderly men. Aging Clin Exp Res. 2004;16(5):375–381. doi: 10.1007/BF03324567 15636463

[2] Luscombe J , Murray AD , Jenkins E , et al. A rapid review to identify physical activity accrued while playing golf. BMJ Open. 2017;7(11):e018993. doi: 10.1136/bmjopen-2017-018993 PMC5719314 29187418

[3] Ainsworth BE , Haskell WL , Herrmann SD , et al. 2011 compendium of physical activities: a second update of codes and MET values. Med Sci Sports Exerc. 2011;43(8):1575–1581. doi: 10.1249/MSS.0b013e31821ece12 21681120

[4] Stevenson EJ , Hayes PR , Allison SJ . The effect of a carbohydrate-caffeine sports drink on simulated golf performance. Appl Physiol Nutr Metab. 2009;34(4):681–688. doi: 10.1139/H09-057 19767804

[5] Burke LM , Hawley JA , Wong SH , et al. Carbohydrates for training and competition. J Sports Sci. 2011;29(Suppl 1):S17–27. doi: 10.1080/02640414.2011.585473 21660838

[6] Hayes LD , Bickerstaff GF , Baker JS . Combined effects of tournament competition and standardisation of pre-performance procedures on salivary testosterone and cortisol concentrations in elite junior golfers. Int J Sports Sci Coach. 2012;7(4):727–738.

[7] Stevenson EJ , Williams C , Mash LE , et al. Influence of high-carbohydrate mixed meals with different glycemic indexes on substrate utilization during subsequent exercise in women. AJCN. 2006;84(2):354–360. doi: 10.1093/ajcn/84.2.354 16895883

[8] Lina BAR , Jonker D , Kozianowski G . Isomaltulose (Palatinose®): a review of biological and toxicological studies. Food Chem Toxicol. 2002;40(10):1375–1381. doi: 10.1016/S0278-6915(02)00105-9 12387299

[9] Nagashima Y , Ehara K , Ehara Y , et al. High-carbohydrate energy intake during a round of golf-maintained blood glucose levels, inhibited energy deficiencies, and prevented fatigue: a randomized, double-blind, parallel group comparison study. Nutrients. 2024;16(23):4120. doi: 10.3390/nu16234120 39683513 PMC11643583

[10] Nagashima Y , Ehara K , Ehara Y , et al. Effects of continuous carbohydrate intake with gummies during the golf round on interstitial glucose, golf performance, and cognitive performance of competitive golfers: a randomized repeated-measures crossover design. Nutrients. 2023;15(14):3245. doi: 10.3390/nu15143245 37513663 PMC10384188

[11] Chambers ES , Bridge MW , Jones DA . Carbohydrate sensing in the human mouth: effects on exercise performance and brain activity. J Physiol. 2009;587(Pt 8):1779–1794. doi: 10.1113/jphysiol.2008.164285 19237430 PMC2683964

[12] Carter JM , Jeukendrup AE , Jones DA . The effect of carbohydrate mouth rinse on 1-h cycle time trial performance. Med Sci Sports Exerc. 2004;36(12):2107–2111. doi: 10.1249/01.MSS.0000147585.65709.6F 15570147

[13] Doan BK , Newton RU , Kraemer WJ , et al. Salivary cortisol, testosterone, and T/C ratio responses during a 36-hole golf competition. Int J Sports Med. 2007;28(6):470–479. doi: 10.1055/s-2006-924557 17111317

[14] Pan X , Soh KG , Jaafar WMW , et al. Mental fatigue in golf: a systematic review. PLoS One. 2025;20(2):e0310403. doi: 10.1371/journal.pone.0310403 39977446 PMC11841881

[15] Mountjoy M , Sundgot-Borgen JK , Burke LM , et al. IOC consensus statement on relative energy deficiency in sport (RED-S): 2018 update. Br J Sports Med. 2018;52(11):687–697. doi: 10.1136/bjsports-2018-099193 29773536

[16] Halson SL . Sleep in elite athletes and nutritional interventions to enhance sleep. Sports Med. 2014;44(Suppl 1):S13–23. doi: 10.1007/s40279-014-0147-0 24791913 PMC4008810

[17] Thomas DT , Erdman KA , Burke LM . Position of the academy of nutrition and dietetics, dietitians of Canada, and the American college of sports Medicine: nutrition and athletic performance. J Acad Nutr Diet. 2016;116(3):501–528. doi: 10.1016/j.jand.2015.12.006 26920240

[18] Thompsett DJ , Vento KA , Der Ananian C , et al. The effects of three different types of macronutrient feedings on golf performance and levels of fatigue and alertness. Nutr Health. 2022;28(4):509–514. doi: 10.1177/02601060221110367 35747933

[19] Jeukendrup A . A step towards personalized sports nutrition: carbohydrate intake during exercise. Sports Med. 2014;44(Suppl 1):S25–33. doi: 10.1007/s40279-014-0148-z 24791914 PMC4008807

[20] Bailey T , Bode BW , Christiansen MP , et al. The performance and usability of a factory-calibrated flash glucose monitoring system. Diabetes Technol Ther. 2015;17(11):787–794. doi: 10.1089/dia.2014.0378 26171659 PMC4649725

[21] Shirtcliff EA , Granger DA , Schwartz E , et al. Use of salivary biomarkers in biobehavioral research: cotton-based sample collection methods can interfere with salivary immunoassay results. Psychoneuroendocrinology. 2001;26(2):165–173. 11087962 10.1016/s0306-4530(00)00042-1

[22] Garde AH , Hansen AM . Long-term stability of salivary cortisol. Scand J Clin Lab Invest. 2005;65(5):433–436. doi: 10.1080/00365510510025773 16081365

[23] Bojkowski CJ , Arendt J , Shih MC , et al. Melatonin secretion in humans assessed by measuring its metabolite, 6-sulfatoxymelatonin. Clin Chem. 1987;33(8):1343–1348. doi: 10.1093/clinchem/33.8.1343 3608151

[24] McCormack HM , Horne DJ , Sheather S . Clinical applications of visual analogue scales: a critical review. Psychol Med. 1988;18(4):1007–1019. doi: 10.1017/S0033291700009934 3078045

[25] Hoddes E , Zarcone V , Smythe H , et al. Quantification of sleepiness: a new approach. Psychophysiology. 1973;10(4):431–436. doi: 10.1111/j.1469-8986.1973.tb00801.x 4719486

[26] Grubbs FE . Procedures for detecting outlying observations in samples. Technometrics. 1969;11(1):1–21. doi: 10.1080/00401706.1969.10490657

[27] Delacre M , Lakens D , Leys C . Why psychologists should by default use Welch's t-test instead of Student's t-test. Int Rev Soc Psychol. 2017;30(1):92–101. doi: 10.5334/irsp.82

[28] Ruxton GD . The unequal variance t-test is an underused alternative to Student's t-test and the Mann–Whitney U test. Behav Ecol. 2006;17(4):688–690. doi: 10.1093/beheco/ark016

[29] Perneger TV . What's wrong with Bonferroni adjustments. BMJ. 1998;316(7139):1236–1238. doi: 10.1136/bmj.316.7139.1236 9553006 PMC1112991

[30] Rothman KJ . No adjustments are needed for multiple comparisons. Epidemiol. 1990;1(1):43–46. doi: 10.1097/00001648-199001000-00010 2081237

[31] Cohen J . Statistical Power Analysis for the Behavioral Sciences. 2nd Hillsdale, NJ: Lawrence Erlbaum Associates; 1988.

[32] Benton D . Carbohydrate ingestion, blood glucose and mood. Neurosci Biobehav Rev. 2002;26(3):293–308. doi: 10.1016/S0149-7634(02)00004-0 12034132

[33] Backhouse SH , Ali A , Biddle SJH , et al. Carbohydrate ingestion during prolonged high-intensity intermittent exercise: impact on affect and perceived exertion. Scand J Med Sci Sports. 2007;17(5):605–610. doi: 10.1111/j.1600-0838.2006.00613.x 17316376

[34] Tassi P , Muzet A . Sleep inertia. Sleep Med Rev. 2000;4(4):341–353. doi: 10.1053/smrv.2000.0098 12531174

[35] Arendt J . Shift work: coping with the biological clock. Occup Med (Lond). 2010;60(1):10–20. doi: 10.1093/occmed/kqp162 20051441

[36] Holub I , Gostner A , Theis S , et al. Novel findings on the metabolic effects of the low glycaemic carbohydrate isomaltulose (Palatinose™). Br J Nutr. 2010;103(12):1730–1737. doi: 10.1017/S0007114509993874 20211041 PMC2943747

[37] Hackney AC . Effects of endurance exercise on the reproductive system of men: the “exercise-hypogonadal Male condition”. J Endocrinol Invest. 2008;31(10):932–938. doi: 10.1007/BF03346444 19092301

[38] Opstad PK . Androgenic hormones during prolonged physical stress, sleep, and energy deficiency. J Clin Endocrinol Metab. 1992;74(5):1176–1183. 1314847 10.1210/jcem.74.5.1314847

[39] Kraemer WJ , Ratamess NA . Hormonal responses and adaptations to resistance exercise and training. Sports Med. 2005;35(4):339–361. doi: 10.2165/00007256-200535040-00004 15831061

[40] Lewis JG . Steroid analysis in saliva: an overview. Clin Biochem Rev. 2006;27(3):139–146. 17268582 PMC1579286

[41] Lennerz BS , Alsop DC , Holsen LM , et al. Effects of dietary glycemic index on brain regions related to reward and craving in men. AJCN. 2013;98(3):641–647. doi: 10.3945/ajcn.113.064113 PMC3743729 23803881

[42] Okuno M , Kim MK , Mizu M , et al. Palatinose-blended sugar compared with sucrose: different effects on insulin sensitivity after 12 weeks supplementation in sedentary adults. Int J Food Sci Nutr. 2010;61(6):643–651. doi: 10.3109/09637481003694576 20367218

[43] König D , Zdzieblik D , Holz A , et al. Substrate utilization and cycling performance following Palatinose™ ingestion: a randomized, double-blind, controlled trial. Nutrients. 2016;8(7):390. doi: 10.3390/nu8070390 27347996 PMC4963866

[44] Jeukendrup AE . Periodized nutrition for athletes. Sports Med. 2017;47(Suppl 1):51–63. doi: 10.1007/s40279-017-0694-2 28332115 PMC5371625

[45] Barnett AG , van der Pols JC , Dobson AJ . Regression to the mean: what it is and how to deal with it. Int J Epidemiol. 2005;34(1):215–220. doi: 10.1093/ije/dyh299 15333621

[46] Garg SK , Smith J , Beatson C , et al. Comparison of accuracy and safety of the SEVEN and the navigator continuous glucose monitoring systems. Diabetes Technol Ther. 2009;11(2):65–72. doi: 10.1089/dia.2008.0109 19243265

[47] Zaharieva DP , Turksoy K , McGaugh SM , et al. Time remains with newer real-time continuous glucose monitoring technology during aerobic exercise in adults living with type 1 diabetes. Diabetes Technol Ther. 2019;21(6):313–321. doi: 10.1089/dia.2018.0364 31059282 PMC6551983

[48] Babiloni C , Del Percio C , Iacoboni M , et al. Golf putt outcomes are predicted by sensorimotor cerebral EEG rhythms. J Physiol. 2008;586(1):131–139. doi: 10.1113/jphysiol.2007.141630 17947315 PMC2375559

[49] Atkinson G , Nevill AM . Selected issues in the design and analysis of sport performance research. J Sports Sci. 2001;19(10):811–827. doi: 10.1080/026404101317015447 11561675

